# Analysis of the Correlation Between Depression-like Behaviors and Lipid Peroxidation in the Prefrontal Cortex of Mice: The Impact of Early Life Stress

**DOI:** 10.3390/brainsci15101112

**Published:** 2025-10-15

**Authors:** Xue Mi, Zi-Ling Ye, Xu-Jun Zhang, Xiao-Man Dai, Zhou-Song Luo

**Affiliations:** 1 Public Technology Service Center, Fujian Medical University, Fuzhou 350122, China; mixue@fjmu.edu.cn; 2 Fujian Key Laboratory of Molecular Neurology, Institute of Neuroscience, Fujian Medical University, Fuzhou 350004, China; yeziling@fjmu.edu.cn (Z.-L.Y.); zhangxujun@fjmu.edu.cn (X.-J.Z.); daixiaoman@fjmu.edu.cn (X.-M.D.)

**Keywords:** early life stress, depression, lipid peroxidation, GPX4, SLC3A2, SLC7A11, correlation

## Abstract

**Objectives**: This study attempted to investigate whether early life stress (ELS) induces lipid peroxidation in the prefrontal cortex (PFC) of mice and its correlation with depression-like behavioral changes. **Methods**: A mouse ELS model was established via maternal separation. Depressive and anxiety-like behaviors were assessed using the forced swim test, sucrose preference test, tail suspension test, and open field test. The expression levels of GPX4, SLC3A2, SLC7A11, TFR1, and lipid peroxidation markers in the PFC of mice were measured and correlated with depression-like behavioral changes. **Results**: ELS induced depressive and anxiety-like behaviors in mice. The mRNA and protein expressions of GPX4, SLC3A2, and SLC7A11 were downregulated in the PFC of ELS mice; the mRNA level of TFR1 was upregulated but its protein level remained unchanged. In the PFC of ELS mice, the product of lipid peroxidation, malondialdehyde, was significantly increased, while the antioxidants, glutathione and superoxide dismutase, were significantly decreased. These markers were significantly correlated with sucrose preference or immobility time of the ELS mice. **Conclusions**: The study evidences that early life stress can induce lipid peroxidation in the PFC of mice and that the latter is closely associated with depression-like behaviors, hinting that ELS may induce depression via lipid peroxidation in the PFC. These findings may suggest a potential strategy for the treatment of early-stage stress-related depression.

## 1. Introduction

As one of the most prominent mood disorders, depression is a major contributor to years of life lost to disability (YLDs) in all age groups except for the group of 0–5 years old. The annual global productivity loss due to depression and anxiety alone is estimated to be USD 1 trillion, while only 9% of people with depression receive adequate treatment [1]. In the disease course of depression, early life stresses (ELSs, also known as adverse childhood experiences in human epidemiological studies) have been closely implicated, including traumatic events, neglect, and abuse. Comprehensive reviews have reported that individuals who experienced ELS are more likely to develop depression before the age of 18 years than those without a history of ELS [2] and that a 10–25% reduction in childhood maltreatment could potentially prevent 31.4–80.3 million cases of depression and anxiety worldwide [3]. Other studies indicate that individuals with a history of ELS are less responsive to treatment [4]. Therefore, due attention should focus on ELS as an important risk factor for depression. Surprisingly, however, little literature is available to elucidate how ELS may change brain structure and function and lead to the development of depression later in life.

Neuroimaging and autopsy reports show that depressed adolescents and adults who have experienced ELS often present significantly reduced gray matter volume and cortical thickness in the prefrontal cortex (PFC) [5], neuronal atrophy, and reduced complexity and density of dendritic spines [6]. Similar phenomena have also been observed in rodent depression models that have undergone maternal separation [7,8]. Although the specific molecular mechanisms have not yet been fully elucidated, increased lipid peroxidation events are documented to be one of the important causes of neuronal atrophy and death [9]. Recent studies have shown that an increase in cerebral lipid peroxidation is observed in patients with depression who have experienced traumatic events in childhood, mice that have experienced maternal separation after birth, and other depression model animals [10,11]. The phenomenon is accompanied by mitochondrial DNA oxidation, mitochondrial membrane depolarization, and increased levels of reactive oxygen species (ROS) [12,13]. Autopsy reports from patients with depression have documented a decrease in antioxidants glutathione (GSH) and glutathione peroxidase in the PFC [14], and patients with major depressive disorder also have high plasma levels of malondialdehyde (MDA, a marker of oxidative stress) [15]. While various antidepressants such as fluoxetine and citalopram can reduce oxidative damage in depressed patients [16], these findings seem to suggest that lipid peroxidation may play an important role in the development of depression in afflicted individuals.

GSH is the primary antioxidant in humans, crucial for maintaining redox balance in mammalian cells. System Xc-, consisting of the protein subunits SLC7A11 and SLC3A2, functions as an amino acid antiporter to facilitate the exchange of extracellular cystine for intracellular glutamate across the plasma membrane, which is crucial for GSH biosynthesis [17]. Targeted inhibition of System Xc- can decrease GSH level and result in GPX4 inactivation, triggering ferroptosis through iron-dependent lipid peroxidation [18,19]. Studies have shown that System Xc-/GPX4 is involved in the progression of chronic social defeat stress (CSDS)-induced depression, which can be ameliorated by edaravone (a free radical scavenger) through the Sirt1/Nrf2/HO-1/Gpx4 axis, thus mitigating neuronal loss and oxidative stress damage in the cortex and hippocampus of mice [20]. Other studies indicate that increasing the expression of System Xc- and GPX4 can reduce ferroptosis in the PFC of type 1 diabetic mice, thereby alleviating their anxiety and depression-like behaviors [21]. Still others suggest that ferroptosis can also be initiated in the hippocampus of depressed mice subjected to chronic unpredictable mild stress [22,23]. However, it remains to be elucidated whether depressed mice that experienced early life stress may feature abnormal changes in the System Xc-/GPX4 antioxidant gene, and whether the depressive behavior of mice is correlated with the lipid peroxidation level in their PFC region.

This study attempted to explore whether ELS would cause changes in antioxidation-related genes and lipid peroxidation indicators in the PFC of mice and to analyze the correlation between these indicators and depression-like behaviors. The results will contribute to the development of potential strategies for ELS-related depression.

## 2. Materials and Methods

### 2.1. Animals

Eight nulliparous female mice and four male mice (8 weeks old) were provided by Guangdong Vital River Laboratory Animal Technology Co., Ltd. (SCXK 2022-0063, Foshan, China). All animals were raised in standard housing conditions with a temperature of 22 ± 2 °C, a relative humidity of 50 ± 10%, a constant light cycle of 12 h light/12 h dark, and unrestricted access to food and water. The Animal Care and Use Ethics Committee of Fujian Medical University rigorously reviewed all animal-related experimental protocols (project identification code: IACUC FJMU 2023-0215; ethics approval date: 8 August 2023). The experimental procedures complied with the European Community’s “Directive on the Care and Use of Laboratory Animals (2010/63/EU)” to uphold animal welfare and ethical standards.

### 2.2. Maternal Separation

Maternal separation was performed using a previously reported method with modifications [24]. After one week of acclimation, mice were bred at a ratio of one male to two females. Males were removed five days after mating, and pregnant females were housed individually until their expected due date (estimated 20–22 days after mating). Littermates of 4–8 pups were included in the experiment. Dams and pups were randomly assigned to either the control or ELS group. Control pups were raised under standard conditions. ELS pups were removed from their cages daily and placed in a 32–34 °C incubator, and their dams were transferred to other clean cages. The dam and her pups were returned to their original cages four hours later. Separation lasted for four hours daily and was continued from postpartum day 1 to postpartum day 21. During this separation period, dams received one-third of their standard nesting material. All cages were changed weekly to minimize disturbance. All pups were weaned at 21 days of age. Male pups were used in the experiment, with 3–5 mice per cage, and raised until adulthood.

### 2.3. Behavioral Tests

Behavioral testing was conducted in a double-blind fashion and initiated when the mice were 8 weeks old. All mice were acclimated to the behavioral testing room for at least 1 h before each test. Behavioral testing was conducted at an interval of 1–2 days. The testing was conducted in an environment of low noise and dim lighting.

#### 2.3.1. Sucrose Preference Test (SPT)

The SPT is used to assess anhedonia-like behavior in mice [25]. The SPT was conducted as previously described [26]. Specifically, mice were housed separately in cages and allowed free drinking from two water bottles for 24 h. Subsequently, one of the bottles was replaced with a 1% sucrose solution, while the other remained unchanged. The drinking habits of the mice were tested for 24 h. To prevent positional preference, the positions of the bottles were switched halfway through the test time. After the test, sucrose consumption was divided by total fluid intake to measure sugar water preference. A decrease in sugar water preference was deemed as an indicator of anhedonia.

#### 2.3.2. Open Field Test (OFT)

The OFT was conducted according to previous reports with slight modification [27]: the dimension of the center area was adjusted from 34 cm × 34 cm as described in the reference to 22 cm × 22 cm in the present study. This adjustment more strictly defined the central zone, placing it closer to the geometric center of the arena, thereby providing a more sensitive measure of anxiety-like behavior in response to early life stresses. Briefly, the OFT was conducted in a white plastic open field arena (44 cm × 44 cm × 40 cm). A center area (22 cm × 22 cm) and four corner areas (11 cm × 11 cm) were mapped on a computer with the SuperMaze software (version 3.0; Shanghai Xinruan Information Tech Co., Shanghai, China). During testing, each mouse was positioned in the arena and permitted to freely explore the area for ten minutes. Its locomotor activity and the time spent in the center and corner areas were recorded. Anxiety-like behaviors were indicated by increased time in the corners or reduced time in the center area.

#### 2.3.3. Forced Swimming Test (FST)

The FST was processed as noted in a previous report [26]. A plastic cylinder (19 cm in diameter × 25 cm in height) was filled with water to a depth of 18 cm and maintained at a temperature of 23–25 °C. The cylinder was placed in an opaque compartment. Mice were placed in the cylinder and swam for 6 min. The behavior of the mice was recorded with SuperMaze software, and the immobility time was automatically calculated. After the test, the mice were dried with a dry towel and returned to their cages. The immobility duration of the mice during the final five minutes was measured. A longer immobility time suggested a depression-like behavior.

#### 2.3.4. Tail Suspension Test (TST)

The TST was performed as previously reported [26]. A 1 cm wide piece of tape was applied 1 cm from the mouse’s tail tip, and the mouse was hung upside down on a metal hook for 6 min. The SuperMaze software was used to automatically record the immobility time. The immobility time for the final five minutes was calculated, with an increase indicating depression-like behavior.

### 2.4. RNA Extraction and Real-Time Quantitative PCR (RT-qPCR)

RNA extraction and RT-qPCR experiments followed a previously established protocol [28]. The initial step involved perfusing the mice with 0.01 M phosphate-buffered saline (PBS). Brain tissue was quickly removed and the PFC was carefully cut out, swiftly frozen in liquid nitrogen, and stored right away at −80 °C. The total RNA was extracted with TriZol reagent (R401-01; Vazyme, Nanjing, China), and the reverse transcription was carried out following standard procedures (R223-01; Vazyme, Nanjing, China). The cDNAs were amplified by RT-qPCR with qPCR SYBR Master (11185ES08, Yeasen, Shanghai, China). The internal control was mouse β-actin, and gene expression was measured by the 2^−ΔΔCT^ method. Table 1 displays the primers used in RT-qPCR.

### 2.5. Western Blot Analysis

As previously described [28], protein levels were assessed using standard Western blot assays. To summarize, cold RIPA buffer (P0013C, Beyotime, Shanghai, China) containing protease and phosphatase inhibitors and PMSF was used to extract brain tissue samples. After resting on ice for 30 min, the samples were centrifuged at 12,000× *g* for 20 min at 4 °C. The supernatant was then collected, and protein levels were measured using the Enhanced BCA Protein Assay Kit (P0010, Beyotime, Shanghai, China) according to the manufacturer’s guidelines. Each sample with 30 μg of protein extract underwent SDS-PAGE, followed by blotting onto a 0.45 μm PVDF membrane in a cold transfer buffer. The membranes were blocked with a blot blocking buffer (P30500, NCM Biotch, Suzhou, China) at room temperature for 1 h and then incubated overnight at 4 °C in specific primary antibodies (GPX4, 1:2000, 67763-1-Ig; SLC3A2, 1:15,000, 15193-1-AP; SLC7A11, 1:5000, 26864-1-AP; TFR1, 1:6000, 10084-2-AP, Proteintech, Wuhan, China. β-actin, 1:10,000, ab8226, Abcam, Cambridge, UK). Subsequently, the membranes were rinsed three times in TBST (10 min per time) and reincubated in a secondary antibody (1:10,000; C31430, C31460, A15999, Invitrogen™, Carlsbad, CA, USA) at room temperature for 60 min. Subsequently, the membranes were rinsed three times in TBST (10 min per time) and detected with the Enhanced ECL Chemiluminescent Substrate Kit (36222ES60, Yeasen, Shanghai, China). The NIH Image J 1.52v software was used to perform grayscale analysis.

### 2.6. Lipid Peroxidation Analysis

MDA, GSH, and superoxide dismutase (SOD) were measured following the instructions provided with the detection kits, respectively (S0131, S0053, S0101, Beyotime, Shanghai, China).

### 2.7. Statistical Analysis

The data were shown as Mean ± SEM and analyzed statistically using GraphPad Prism 9.0 (GraphPad Software, Boston, CA, USA). Differences between groups were evaluated by an unpaired two-sample two-tailed *t*-test. In the correlation analysis, data normality was assessed by the Shapiro–Wilk test. Data with a normal distribution were evaluated via Pearson correlation analysis, with the results reported with the Pearson correlation coefficient (*r*) and the corresponding *p*-value. Data without a normal distribution were processed via Spearman rank correlation analysis, with the results depicted with the Spearman rank correlation coefficient (*ρ*) and the corresponding *p*-value. Data beyond the range of ±2-fold SD were considered as an abnormality and excluded from analyses. Statistical significance was set at *p* < 0.05.

## 3. Results

### 3.1. ELS-Induced Depression- and Anxiety-like Behaviors in Mice

**Figure 1:**
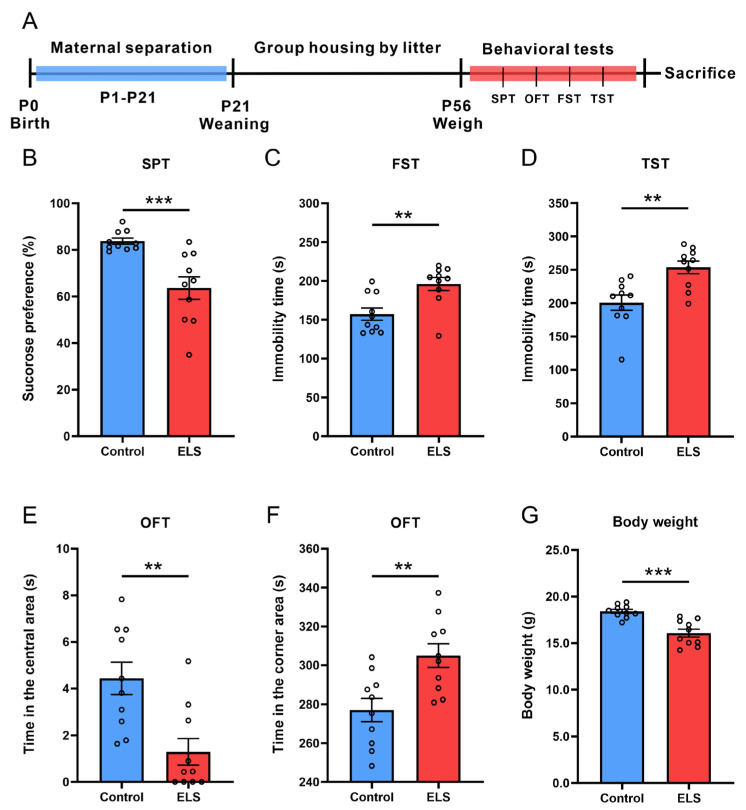
ELS induction of depression- and anxiety-like behaviors in mice accompanied by weight loss. (**A**) Schematic diagram of the animal experiment. (**B**) Sucrose preference of mice in the SPT. (**C**) Immobility time of mice in the FST. (**D**) Immobility time of mice in the TST. (**E**) Time spent in the central area of the OFT. (**F**) Time spent in the corner area of the OFT. (**G**) The body weight. Data are expressed as the mean ± SEM, *n* = 10 per group. ** *p* < 0.01; *** *p* < 0.001, as compared with the control group.

The experimental design is depicted in Figure 1A. The SPT, FST, and TST were used to evaluate depression-like behaviors in mice (Figure 1B–D). In the SPT, the ELS group showed a notably lower sucrose preference when compared with the control group (*p* = 0.0008) (Figure 1B). In the FST and TST, immobility time was significantly longer in the ELS group than in the control group (*p* = 0.0035; *p* = 0.0022, respectively) (Figure 1C,D). Anxiety-like behaviors in mice were assessed in the OFT (Figure 1E,F). The results showed that, compared with the control group, the ELS group spent significantly less time in the central area (*p* = 0.0026) and more time in the corner areas (*p* = 0.0041). These data indicate that ELS successfully induces depression- and anxiety-like behaviors in mice. Furthermore, the ELS group weighed significantly less than the control group (*p* = 0.0001) (Figure 1G), likely due to early life separation from their mothers, which limited their free nutrient intake.

### 3.2. Correlation Analysis Between the mRNA Expression of Antioxidation Genes and Depression-like Behavior in Mice

**Figure 2:**
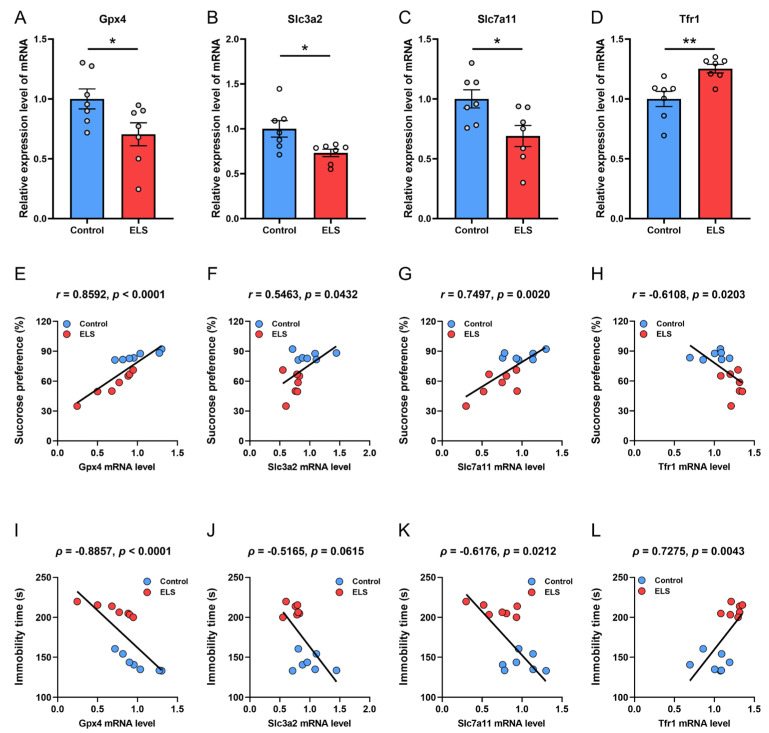
The mRNA level of Gpx4, Slc3a2, Slc7a11, and Tfr1 of the PFC and correlation analysis with depression-like behaviors in the ELS model. (**A**–**D**) The relative mRNA level of (**A**) Gpx4, (**B**) Slc3a2, (**C**) Slc7a11, and (**D**) Tfr1 in the PFC. Data are expressed as the mean ± SEM, *n* = 7 per group. * *p* < 0.05; ** *p* < 0.01, as compared with the control group. (**E**–**H**) Pearson correlation analysis between (**E**) Gpx4, (**F**) Slc3a2, (**G**) Slc7a11, (**H**) Tfr1, and sucrose preference of SPT. (**I**–**L**) Spearman rank correlation analysis between (**I**) Gpx4, (**J**) Slc3a2, (**K**) Slc7a11, and (**L**) Tfr1, and immobility time of FST.

We examined the expression of several key anti-lipid oxidation genes, including Gpx4, Slc3a2, Slc7a11, and the transferrin receptor Tfr1 (Figure 2A–D). We found that after maternal separation, the mRNA expressions of Gpx4, Slc3a2, and Slc7a11 in the PFC of mice were significantly lower than those in control mice (*p* = 0.0386; *p* = 0.0207; *p* = 0.0208, respectively) (Figure 2A–C), while the mRNA level of Tfr1 was significantly higher than that of the control mice (*p* = 0. 0047) (Figure 2D). We then analyzed the correlation between gene expression and mouse behavior (Figure 2E–L). Pearson correlation analysis examined the relationship between mRNA levels of Gpx4, Slc3a2, Slc7a11, and Trf1 in the PFC of mice and the degree of sucrose preference during SPT (Figure 2E–H). The findings indicated a significant positive correlation between sucrose preference and the mRNA expression levels of Gpx4 (*r* = 0.8592, *p* < 0.0001), Slc3a2 (*r* = 0.5463, *p* = 0.0432), and Slc7a11 (*r* = 0.7497, *p* = 0.0020); the mRNA expression of Tfr1 was significantly negatively correlated with sucrose preference (*r* = −0.6108, *p* = 0.0203). Spearman correlation analysis was employed to assess the correlation between the above genes and immobility time on the FST (Figure 2I–L). The results indicated a significant negative correlation between mRNA expression of Gpx4 (*ρ* = −0.8857, *p* < 0.0001) (Figure 2I) and Slc7a11 (*ρ* = −0.6176, *p* = 0.0212) (Figure 2K) with immobility time in the FST. The mRNA expression of Tfr1 was significantly positively correlated with the immobility time on the FST (*ρ* = 0.7275, *p* = 0.0043) (Figure 2L). There was no significant correlation found between SLC3A2 mRNA expression and immobility time in the FST (*p* = 0.0615) (Figure 2J). These results suggest that changes in the mRNA levels of these genes regulating lipid peroxidation may be an important factor influencing the depression-like behavior induced by early stress in mice.

### 3.3. Correlation Analysis Between the Protein Expression of Antioxidation Genes and Depression-like Behavior in Mice

**Figure 3:**
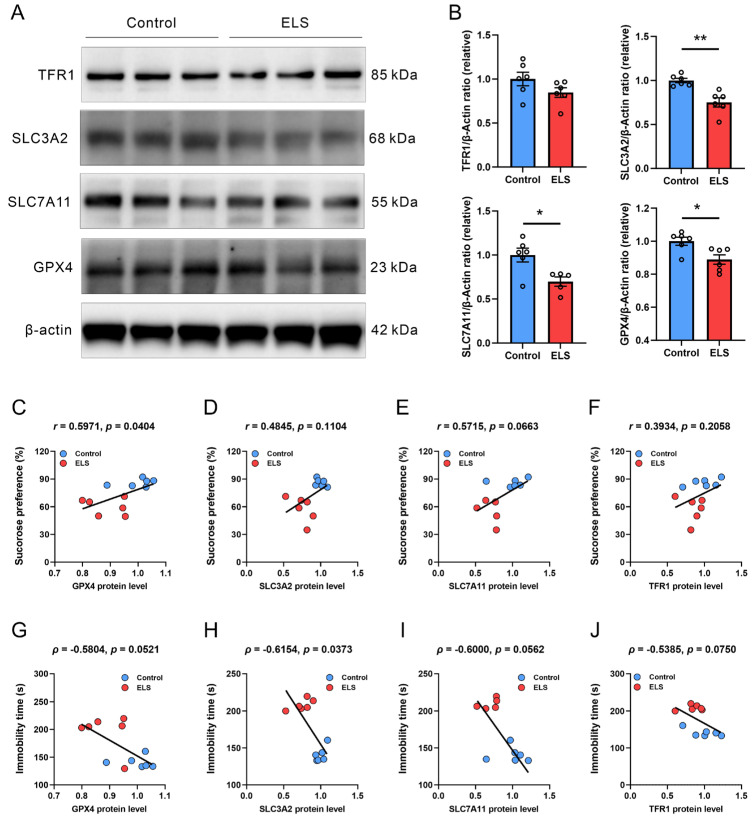
The protein level of GPX4, SLC3A2, SLC7A11, and TFR1 of the PFC and their correlation with depression-like behavior in the ELS model. (**A**) Representative original Western blot bands and (**B**) the relative protein expression levels of GPX4, SLC3A2, SLC7A11, and TFR1 in the PFC. Data are expressed as the mean ± SEM, *n* = 6 per group. * *p* < 0.05; ** *p* < 0.01, as compared with the control group. (**C**–**F**) Pearson correlation analysis between (**C**) GPX4, (**D**) SLC3A2, (**E**) SLC7A11, (**F**) TFR1, and sucrose preference of SPT. (**G**–**J**) Spearman rank correlation analysis between (**G**) GPX4, (**H**) SLC3A2, (**I**) SLC7A11, (**J**) TFR1, and immobility time of FST.

We analyzed the expression levels of the aforementioned anti-lipid oxidation proteins (Figure 3A,B). We found that the protein levels of GPX4, SLC3A2, and SLC7A11 in the PFC of ELS mice were significantly lower (*p* = 0.0144; *p* = 0.0016; *p* = 0.0139, respectively) when compared with those of the control mice. There was no notable difference in TFR1 protein levels between the control and ELS groups. We then analyzed the correlation between protein expression and mouse behavior (Figure 3C–J). Pearson correlation analysis was employed to investigate the correlation between the protein expression levels of GPX4, SLC3A2, SLC7A11, and TFR1 in the PFC of mice and the sucrose preference rate during SPT (Figure 3C–J). The data revealed that the protein expression of GPX4 (*r* = 0.5971, *p* = 0.0404) was significantly positively correlated with the sucrose preference of mice (Figure 3C). However, the protein expression of SLC3A2, SLC7A11, and TFR1 was not significantly correlated with the sucrose preference rate of mice (Figure 3D–F). Spearman correlation analysis of the correlation between the aforementioned genes and immobility time in the FST (Figure 3G–J) revealed that the protein expression of SLC3A2 (*ρ* = −0.6154, *p* = 0.0373) (Figure 3H) was significantly negatively correlated with immobility time in the FST. However, there was no significant correlation between protein expressions of GPX4, SLC3A2, and TFR1 and the immobility time in the FST (Figure 3G,I,J). These results suggest that, at the protein level, the regulation of depression-like behavior in mice by different anti-lipid peroxidation genes may be behaviorally specific.

### 3.4. Correlation Analysis Between the Level of Lipid Peroxidation in the PFC and Behavior in ELS Mice

**Figure 4:**
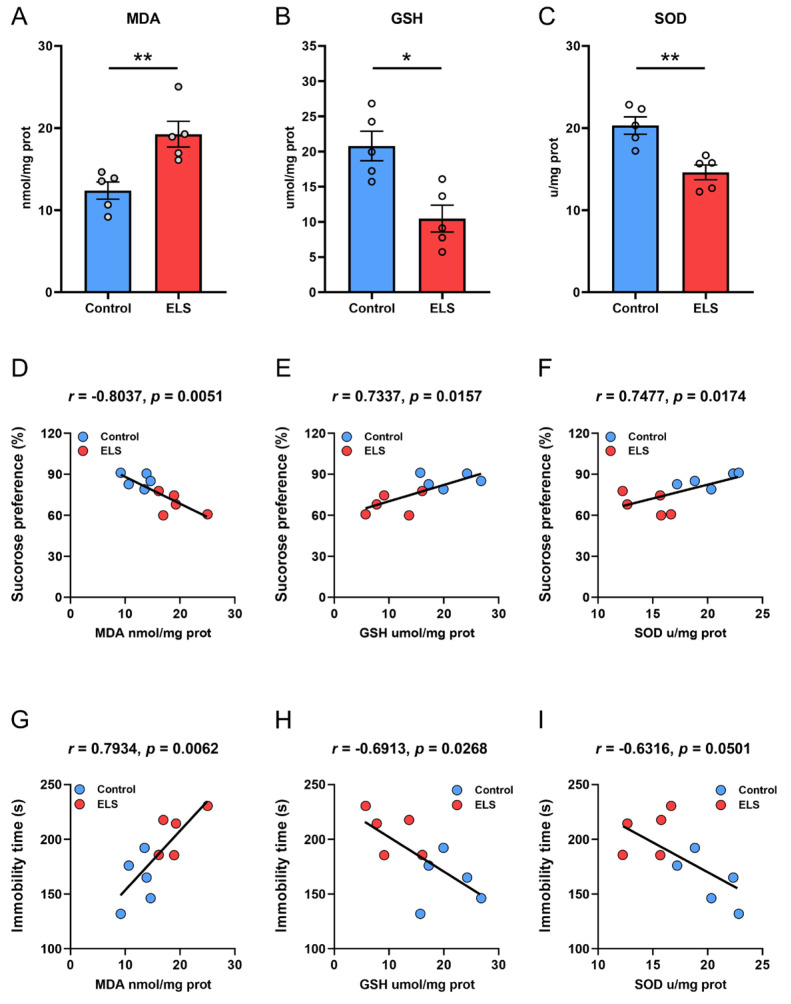
Lipid peroxidation levels in the PFC and correlation analysis with depression-like behavior in the ELS model. (**A**–**C**) The level of (**A**) MDA, (**B**) GSH and (**C**) SOD in the PFC. Data are expressed as mean ± SEM, *n* = 5 per group. * *p* < 0.05; ** *p* < 0.01, as compared with the control group. (**D**–**F**) Pearson correlation analysis between (**D**) MDA, (**E**) GSH, and (**F**) SOD, and sucrose preference of SPT. (**G**–**I**) Pearson correlation analysis between (**G**) MDA, (**H**) GSH, and (**I**) SOD, and immobility time of FST.

Finally, we examined the level of lipid peroxidation in the PFC of mice. The results showed that compared with the control group, mice experiencing maternal separation reported a significant increase in MDA levels in the PFC (*p* = 0.0064) (Figure 4A) but a marked decrease in antioxidant GSH and SOD levels (*p* = 0.0066; *p* = 0.0034, respectively) (Figure 4B,C). We then used Pearson correlation to assess the correlation between levels of MDA, GSH, and SOD and SPT. The results showed that MDA (*r* = −0.8037, *p* = 0.0051) (Figure 4D) was significantly negatively correlated with sugar water preference, while GSH (*r* = 0.7337, *p* = 0.0157) (Figure 4E) and SOD (*r* = 0.7455, *p* = 0.0174) (Figure 4F) had a strong negative correlation with sugar water preference. Pearson correlation analysis of the correlation between the level of lipid peroxidation in the PFC and immobility time in the FST showed that MDA (*r* = 0.7934, *p* = 0.0062) (Figure 4G) was significantly positively correlated with immobility time; GSH (*r* = −0.6913, *p* = 0.0268) (Figure 4H) was significantly negatively correlated with the immobility time; and the level of SOD (Figure 4I) was not significantly correlated with the immobility time in FST. These results show that the increased degree of lipid peroxidation and the weakened antioxidant capacity of mice are significantly associated with the aggravation of depression-like behaviors (loss of pleasure and despair-like behaviors).

## 4. Discussion

This study investigated the effects of ELS on depression-like behaviors in adult mice, the expression of anti-lipid peroxidation genes in the PFC, and the level of lipid peroxidation, and verified the correlations between these key indicators. The results showed that ELS-treated mice exhibited significant depression- and anxiety-like behaviors in adulthood. The levels of antioxidation genes (GPX4, SLC3A2, and SLC7A11) in the PFC were significantly downregulated, while TFR1 level was upregulated. This was accompanied by an elevation in the lipid peroxidation product, MDA, and a decline in the antioxidants, GSH and SOD. Furthermore, these molecular markers showed a significant correlation with depression-like behaviors, including SPT and FST. This study is the first attempt to systematically depict the “ELS-lipid peroxidation-depressive behavior” link in the ELS model, providing experimental evidence for the lipid peroxidation mechanism underlying ELS-induced depression and a theoretical basis for targeting lipid peroxidation in managing ELS-related depression.

Epidemiological studies have revealed that individuals who experience ELSs are more likely to develop mood-related disorders such as depression and anxiety [2,29,30,31,32]. An epidemiological study of 9508 people reports that compared with those without ELSs exposure, individuals exposed to four or more types of childhood ELS experiences face a 4- to 12-fold higher risk of alcoholism, drug addiction, depression, and suicide attempts [29]. In addition, childhood abuse is associated with an increased risk of recurrent and persistent depression in adulthood, and with nonresponse or relapse during depression treatment [4]. This suggests that ELSs have a significant impact not only on the onset of depression but also on its treatment. In the current study, we successfully replicated the classic model of ELS-induced depression-like behaviors in mice. The results showed that ELS mice exhibited typical depression-like behaviors in adulthood: a decrease in sucrose preference indicating anhedonia, a prolonged immobility in the FST and TST reflecting despair-like behavior, and a decreased exploration of the central area and increased time spent in corners in the OFT reflecting anxiety-like emotions. This is highly consistent with prior studies of the ELS model [26,33,34].

In addition, the ELS mice had a notably lower weight when compared with the control group, which is directly related to the restricted early nutritional intake. Human data have indicated early malnutrition as an independent risk factor for an increased risk of depression later in life. During adolescence (11–17 years old), even though those who were previously malnourished are not physically different from healthy children, they present a significantly higher level of depressive symptoms [35]. In our study, the changes in behavioral and growth indicators not only confirmed a successful establishment of the ELS model but also suggested that ELS, as an intense stressor, has long-lasting and harmful effects on the central nervous system of mice. ELS produces a coordinated damage of multiple systems in mice, which echoes the clinical phenomenon that people with early adverse experiences face an increased risk of depression and other chronic diseases [29,36,37,38].

One of the key findings of this study is that both mRNA and protein levels of GPX4, SLC3A2, and SLC7A11 were significantly downregulated in the PFC of ELS mice, and that some of these genes were specifically associated with depression-like behaviors. This is the first link between ELS and lipid peroxidation in the PFC. The glutathione peroxidase family comprises numerous members, forming a vast enzyme system. A hallmark of this family is the presence of a cysteine residue at its catalytic site that acts as a redox-active center—this structural feature enables the enzyme to mediate oxidation-reduction reactions [39]. As the most important member of the GPX family, GPX4 is an antioxidant enzyme that directly reduces phospholipid peroxides in membranes and lipoproteins [40] and is essential for cell survival and mammalian embryonic development [41]. Compared with other GPX isoforms, GPX4 has a broad substrate specificity [42]. A downregulation of GPX4 expression can directly reduce the efficiency of lipid peroxide clearance. GSH is the reducing substrate for GPX4 activity, and GPX4 catalyzes the reduction of lipid hydroperoxides by oxidizing GSH to glutathione disulfide, which is essential for GPX4 function [43]. The cystine-glutamate transporter (xCT), composed of SLC3A2 and SLC7A11, is a key carrier for GSH synthesis. A downregulation of both can reduce cystine uptake, leading to an insufficient GSH synthesis [44]. Therefore, the downregulation of these genes in the PFC region of mice experiencing ELS can result in a dual antioxidant defect of “decreased clearance ability and insufficient synthesis materials”, which lays the groundwork for the subsequent aggravation of lipid peroxidation.

The available literature has reported that the disruption of iron balance in the body is associated with a variety of neurological diseases [45]. Excessive iron ions can greatly accelerate fatty acid peroxidation, leading to the continuous accumulation of lipid peroxides. When the resulting lipid ROS exceeds the tolerance of the cellular antioxidant system, cell death ultimately ensues, a process known as ferroptosis [46]. In the current study, the mRNA level of TFR1, an iron uptake receptor, was upregulated in the ELS mice, indicating an increased potential for iron uptake in the PFC. Iron overload is a necessary condition for ferroptosis [47]. In the current study, in terms of the correlation between mRNA levels and depressive behaviors, the mRNA expression of GPX4, SLC3A2, and SLC7A11 was positively associated with sucrose preference and negatively correlated with immobility time (GPX4 and SLC7A11), while the mRNA level of TFR1 reported an inverse correlation with the two behavioral measures. This correlation pattern is highly consistent with the functions of these genes. GPX4 is a core enzyme for scavenging lipid peroxides, while SLC3A2 and SLC7A11 form System Xc-, which is essential for GSH synthesis. A downregulation of these three genes directly impairs antioxidant capacity in the PFC, leading to the accumulation of lipid peroxidation products [19]. These genes are associated with both sugar preference, reflecting anhedonia, and immobility time, reflecting despair-like behaviors, suggesting that oxidative damage may play a role in regulating reward perception and despair. These results indicate that ELS may push PFC cells into a vulnerable state prone to ferroptosis by disrupting the System Xc-/GPX4 antioxidant axis and promoting iron uptake.

Notably, the correlations at the protein level revealed a significant behavioral specificity: only the protein level of GPX4 showed a positive correlation with sucrose preference in the SPT, while the protein level of SLC3A2 was negatively associated with immobility time in the FST. No significant correlations were detected for SLC7A11 or TFR1 proteins. This discrepancy between broad mRNA correlations and specific protein associations may stem from differences in post-translational regulation of gene expression. GPX4, the most potent antioxidant enzyme in the body, directly determines the resistance of the PFC to lipid peroxidation damage. The current study revealed a strong positive correlation of both its mRNA and protein expression levels with sucrose preference, suggesting a possible role in regulating the reward system. As for SLC3A2, which forms the cystine/glutamate antiporter with SLC7A11, its reduction directly limits the efficiency of cystine uptake, affecting GSH synthesis and, consequently, the resistance to lipid peroxidation [48]. Furthermore, as this transporter takes up cystine, it releases glutamate into the extracellular space [49]. A decrease in SLC3A2 protein level can also disrupt the stability of glutamate concentrations, affecting neuronal excitability. These two factors may jointly play a role in regulating despair in mice.

Previous studies have reported increased lipid peroxides in major depression [50], melancholia [51], treatment-resistant depression [10], and comorbid major depression with generalized anxiety disorder [52]. We measured MDA, GSH, and SOD levels and confirmed that ELS induces an imbalance in lipid peroxidation in the PFC, which is significantly correlated with depression-like behaviors, providing preliminary support for the causal relationship of “early stress-lipid peroxidation-depression.” In particular, mice in the ELS group exhibited a notable rise in MDA levels when compared with the control group, suggesting an accumulation of lipid peroxidation products, while GSH and SOD levels were significantly decreased, indicating impaired antioxidant systems, consistent with the effect conferred by a downregulated expression of GPX4 and SLC3A2/SLC7A11. The correlation analysis showed that MDA level had a negative correlation with sucrose preference and a positive correlation with immobility time in the FST, while GSH and SOD levels were inversely correlated, indicating that the degree of lipid peroxidation is highly correlated with the severity of ELS-induced depression-like behaviors. Although low levels of lipid peroxidation products can trigger protective cellular signaling and boost antioxidant defenses [53], excessive levels may produce harmful effects, as has been widely demonstrated in neurological diseases [10,54,55,56,57]. Lipid peroxides can impair PFC function through multiple pathways. For example, lipid peroxidation can result in oxidative damage to cellular molecules such as DNA, proteins, and lipids, inducing membrane disorganization and functional loss [58]. Lipid peroxidation-induced damage to neurons may lead to reduced release of neurotransmitters, thereby affecting signaling in reward and mood regulation pathways [59,60,61]. An increase in lipid peroxides can also disrupt blood–brain barrier function and upregulate the inflammatory mediators, further exacerbating neuronal damage [62]. These results are consistent with recent findings that oxidative stress is involved in the pathology of depression [63,64].

## 5. Significance and Limitations

This study, in the context of ELS, a key environmental risk factor, reveals the central role of PFC lipid peroxidation in depression susceptibility among adults. Although lipid peroxidation has been extensively reported in various depression models, existing studies primarily focus on mechanistic exploration or pharmacological interventions and have largely overlooked the potential direct correlations between specific molecular alterations and behavioral phenotypes, particularly in ELS. This study, for the first time, observed coordinated changes in key lipid peroxidation regulatory genes in the PFC, including GPX4/SLC7A11/SLC3A2/TFR1, in the ELS model. Importantly, these molecular alterations were significantly associated with depression-like behaviors in adulthood, providing strong behavioral-molecular evidence for the hypothesis that lipid peroxidation is a key intermediate in ELS-induced depression-like behaviors. Therefore, the significance of this study transcends the descriptive observations and shifts mechanistic research in the ELS-induced depression model to behavioral validation, establishing, for the first time, the critical role of the lipid peroxidation pathway in this ELS model. This finding not only deepens our understanding of the long-term effects of ELS on mood but also provides a unique theoretical basis and potential target for targeting lipid peroxidation in the treatment of ELS-induced depression subtypes.

Of course, this study primarily revealed correlations, which still need to be tested and furthered in broader research dimensions. Several limitations remain. First, this study only confirmed the association between genes, lipid peroxidation, and behavior by the correlation analysis; intervention studies (e.g., gene overexpression or knockdown) have not yet been conducted to verify whether these molecules are essential for ELS-induced depression. Second, this study examined genetic and biochemical markers in the entire PFC tissue, unable to distinguish the contributions of different cell types, such as neurons, astrocytes, and microglia. Further exploration of cell-specific mechanisms is needed. Third, in addition to the PFC, the hippocampus is also a brain region sensitive to early stress. Further investigations are needed to investigate whether similar abnormalities of lipid peroxidation are present in other brain regions in order to refine the mechanistic network. Fourth, maternal separation is only one type of ELS model, and depression also has obvious sex differences, so the pathological mechanisms of different models and sexes may be different. Fifth, this study only focused on the role of lipid peroxidation in ELS-induced depression-like behaviors. However, depressed patients often have complex clinical manifestations, such as fluctuations in appetite, which may involve mechanisms such as gut microbial disorders and neurotransmitter imbalances. Whether these concerns are correlated with ELS-induced behavioral changes is also a valuable research direction. Future research can explore the possibility of blocking the pathological process of depression caused by early stress through in-depth research using technologies such as genetically modified mice, virus-mediated PFC-specific gene regulation, and single-cell sequencing technology, and provide new theoretical targets and experimental basis for the development of preventive antidepressant strategies for “people at high risk of ELS”.

## 6. Conclusions

This study evidences that ELS may downregulate the expression of GPX4, SLC3A2, and SLC7A11 in the PFC of mice undergoing early adverse experiences, inducing lipid peroxidation imbalance (increased MDA and decreased GSH/SOD), and that these indicators are significantly correlated with depression-like behaviors. This study suggests that ELS may induce depression through lipid peroxidation in the PFC, providing a potential strategy for intervening in early-stage stress-related depression.

## Figures and Tables

**Table 1:** Primers of the genes investigated in the RT-qPCR analysis.

Gene		Primer Sequences
Gpx4	F	GATGGAGCCCATTCCTGAACC
	R	CCCTGTACTTATCCAGGCAGA
Slc3a2	F	GGTGCTCAACTTCCGAGATTC
	R	TCATAAGGATTCAGGCTCAGGTT
Slc7a11	F	AGGGCATACTCCAGAACACG
	R	GGACCAAAGACCTCCAGAATG
Tfr1	F	CAGCAAAGTCTGGCGAGATG
	R	GAATGCCACATAACCCTCGG
β-actin	F	TGTCCACCTTCCAGCAGATGT
	R	AGCTCAGTAACAGTCCGCCTAG

## Data Availability

The original contributions presented in this study are included in the article. Further inquiries can be directed to the corresponding author.

## Abbreviations

The following abbreviations are used in this manuscript:

ACEs	Adverse childhood experiences
CSDS	Chronic social defeat stress
ELS	Early life stress
FST	Forced swim test
GSH	Glutathione
MDA	Malondialdehyde
OFT	Open field test
PBS	Phosphate-buffered saline
PFC	Prefrontal cortex
ROS	Reactive oxygen species
RT-qPCR	Real-time quantitative PCR
SOD	Superoxide dismutase
SPT	Sucrose preference test
TST	Tail suspension test
YLDs	Years of life lost to disability
